# Atomistic Investigation on Diffusion Welding between Stainless Steel and Pure Ni Based on Molecular Dynamics Simulation

**DOI:** 10.3390/ma11101957

**Published:** 2018-10-12

**Authors:** Yanqiu Zhang, Shuyong Jiang

**Affiliations:** College of Mechanical and Electrical Engineering, Harbin Engineering University, Harbin 150001, China; zhangyq@hrbeu.edu.cn

**Keywords:** diffusion welding, molecular dynamics, interface, roughness, pressure

## Abstract

Based on molecular dynamics (MD) simulation, the behaviors and mechanisms of diffusion welding between 304 stainless steel (304 SS) and pure Ni were investigated in the present study. The results show that surface roughness has a significant influence on the diffusion behaviors of atoms during diffusion welding between two different materials, and it is suggested that the rough surface should be set on the pure Ni rather than the 304 SS during the diffusion welding between them. Temperature plays an important role in the interface diffusion. With the increase of temperature, the number of atoms diffusing into the opposite side increases and the diffusion distances increase as well. As a consequence, the diffusion welding should be performed at a suitably elevated temperature. The influence of vertical pressure on the diffusion bonding between the two materials includes two aspects. One is to increase the contact area via deforming the asperities or grooves at the interface, which provides more opportunities for the diffusion between the two materials. The other is to reduce the mobility of atoms within a lattice. As a consequence, the pressure effect is smaller than temperature effect during diffusion welding between 304 SS and pure Ni.

## 1. Introduction

Diffusion welding is a method which realizes the coalescence of surfaces of two parts by means atom diffusion in the condition of a certain temperature and a certain vertical pressure. This welding method has been widely applied in the fields of aviation, aerospace, instruments and electronics because it results in compatible chemical components and perfect properties at the joints [1,2]. So far, numerous theoretical and experimental investigations have been performed on the diffusion welding [3,4,5,6,7,8]. However, much of these studies focused on the microstructures and properties of the joints, and little literatures related to the diffusion mechanisms and behaviors of the interface have been found because the atomic diffusion during the welding is difficult to be observed by means of conventional methods.

Molecular dynamics (MD) simulation provides a powerful method to study the material behaviors at atomistic scale [9,10,11,12]. In recent years, MD simulation has been widely applied to investigate the behaviors and mechanisms of atom diffusion at atomistic scale. Evteev et al. investigated diffusion behavior of Ni vacancies and Ni antisite defects in B2-NiAl [13]. Lu et al. investigated the diffusion behaviors of hydrogen atoms in body-centered cubic (BCC) Fe containing point defects [14]. Bai et al. investigated the diffusion behaviors of the interface between the tool and the chip during machining titanium alloy [15]. Song et al. studied the atomic diffusion behaviors in linear friction welding between Ti and Ti–Al alloy [16]. Luo et al. investigated the diffusion phenomenon at Mo–Ti interface, and they found that the temperature plays a key role in the course of interface diffusion [17].

However, all of the aforementioned literature focused on the atom diffusion which occurs at the interface between two materials containing one or two elements. In fact, the practical joints are often composed of alloys which possess several elements. Consequently, the diffusion in these interfaces becomes more complicated. So far, no literature related to the diffusion among three elements has been found. In particular, no literature concerning the MD simulation of diffusion welding between multi-component alloy and pure metal have been found. The present study aims to investigate the behaviors and mechanisms of diffusion welding between 304 stainless steel (304 SS) and pure Ni based on MD simulation, where the 304 SS is composed of 74% Fe, 18% Cr and 8% Ni (wt.%).

## 2. Modeling and Method

The MD simulation was conducted via LAMMPS package developed by Plimpton [18]. Interatomic potential plays a crucial role in a multi-component alloy and it has a significant influence on the results of MD simulations. In the present study, the embedded atom method (EAM) potential developed by Bonny et al. [19] was adopted to describe the interactions among atoms of 304 SS. The potential was established for face-centered cubic (FCC) FeNiCr alloy, where the total energy can be expressed as

(1) $$E=\frac{1}{2}\sum_{\begin{array}{l} i,j=1\\ i\neq j \end{array}}^{N}V_{t_{i}t_{j}}(r_{ij})+\sum_{i=1}^{N}F_{t_{i}}(\rho_{i})\;$$

where V is the pair interaction, and F is the embedding energy, which is dependent on the local electron density ρ. The latter term of Equation (1) is similar to the many-body contribution provided by all neighbouring atoms. In Equation (1), N denotes the total number of atoms in the system, $r_{ij}$ represents the distance between atoms *i* and *j*, and $t_{i}$ is chemical species (Fe, Ni or Cr in the present case). The local electron density $\rho_{i}$, which is contributed by the neighbors around atom *i*, is able to be obtained by

(2) $$\rho_{i}=\sum_{\begin{array}{l} j=1\\ j\neq i \end{array}}^{N}\phi_{t_{j}}(r_{ij})\;$$

where ϕ is the electron density function of the considered element.

As the potential of a multi-component alloy can be used for a binary alloy or a pure phase, the above potential was also suitable to describe the interactions among atoms of pure Ni in the present study so that the diffusion laws of Ni atoms in pure Ni are in accordance with the ones in 304 SS.

**Figure 1:**
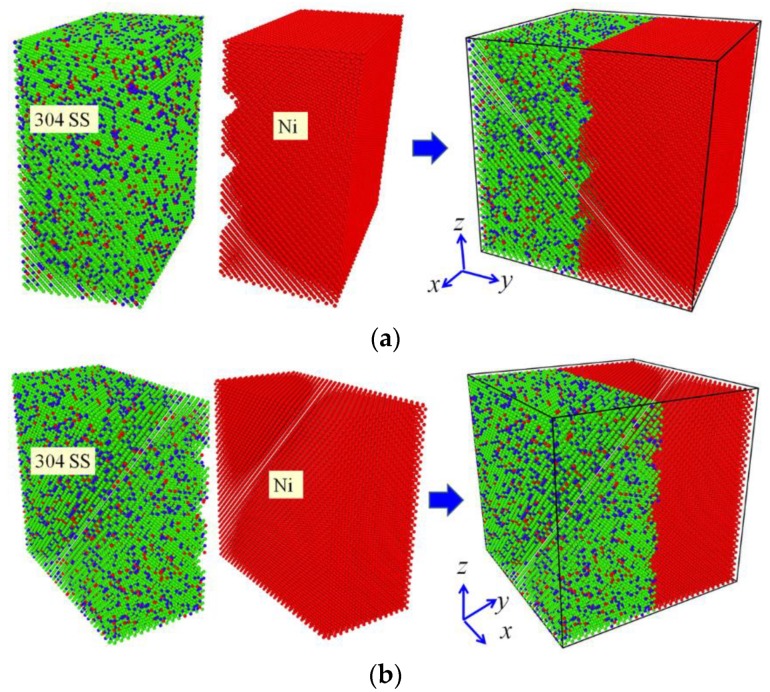
Molecular dynamics (MD) simulation models for the diffusion welding: (**a**) Model Ι; (**b**) Model ΙΙ. (In the models, Fe atoms are presented by green color, Ni atoms are denoted by red color and Cr atoms are exhibited by blue color).

**Figure 2:**
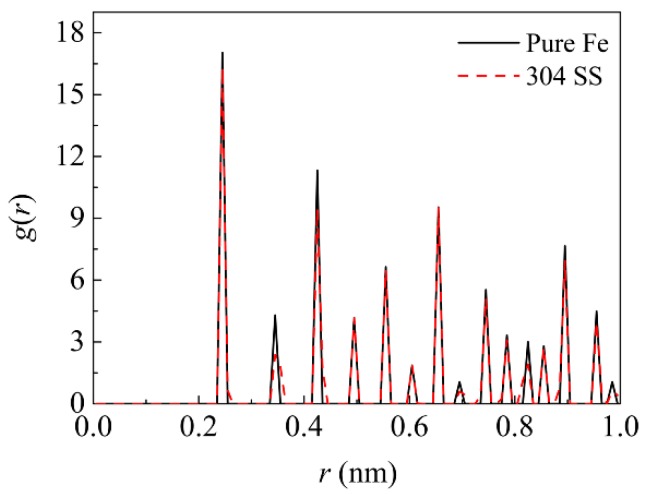
Comparison of radial distribution function (RDF) between the pure Fe and 304 stainless steel (304 SS).

Previous experiments have demonstrated that the roughness of contact surfaces has a significant influence on the bonding process of diffusion welding [20,21,22,23]. However, these studies have not been undertaken in a case that the same roughness is set on the opposite materials alternately. In the present study, two MD simulation models for the diffusion welding were prepared so as to study the influence of roughness, as illustrated in Figure 1. Both the models have the size of 10.496 × 10.496 × 10.496 nm and they are both composed of two parts, where the left half is the 304 SS with the composition of 74% Fe, 18% Cr and 8% Ni (wt.%), and the right half is pure Ni. In the case of model Ι, three half cylindrical grooves with the radius of 0.5 nm were set on the interface surface of the pure Ni. In the case of model ΙΙ, three half cylindrical grooves with the same size were set on the interface surface of 304 SS. The distance between the centers of two nearest grooves was set as 2.624 nm in both models and the centers of these grooves were set at the interface, where the middle one is located in the half height of the models. Both the 304 SS and the pure Ni possess the FCC structure, and their lattice constants are 0.35 nm and 0.352 nm, respectively. In the simulation models, the [100], [010] and [001] directions of the both halves were parallel to the x, y and z directions of Cartesian coordinate system, respectively. The structure of 304 SS was established such that perfect FCC Fe atoms was firstly filled in the left half of the box, and subsequently Cr atoms with the fraction of 18% (wt.%) and Ni atoms with the fraction of 8% (wt.%) were filled in the box by substituting the Fe atoms randomly. These Cr and Ni atoms were located in the positions where the Fe atoms had been laid. The substituted Fe atoms were selected randomly rather than being selected in definite positions. As a consequence, the generated structure type remained unchanged, but it was transformed from a pure metal into an alloy. Figure 2 illustrates the comparison of radial distribution function (RDF) between the pure Fe and 304 SS. It can be seen that the two metals still present the same structure type except that the width or height of some peaks is slightly altered after the alloying. This difference is attributed to the fact that the substitution of Ni and Cr atoms induces the lattice distortion of Fe matrix due to the size difference among the three elements.

During the simulation, periodic boundaries were set in all the three directions. Before the models were subjected to any load, relaxations at 0.01 K were performed on them based on conjugate gradient method so that the energy of these systems could be minimized. Subsequently, the models were heated from 0.01 K to the considered temperatures in 10 ps and then they were held at the temperature for 200 ps. During the heating and holding time, a definite vertical pressure was applied on the models in *y* direction. In order to investigate the influences of temperature and vertical pressure on the diffusion behaviors of interface during diffusion welding, three temperatures (1000, 1200 and 1400 K) and three vertical pressures (0.5, 5 and 50 MPa) were considered in the present study.

In order to provide the information for diffusion behaviors of atoms during diffusion welding, the calculation of mean square displacement (MSD) was conducted in the current investigation. According to the definition, MSD can be expressed as [16]

(3) $$r^{2}\left(t\right)=\frac{1}{N}\left\{\sum_{i=1}^{N}{\left|{\vec{r}}_{i}\left(t\right)-{\vec{r}}_{i}\left(0\right)\right|}^{2}\right\}\;$$

where *N* is the total number of atoms in the system, and ${\vec{r}}_{i}\left(t\right)$ denotes the position of atom *i* at time *t*.

## 3. Results and Discussion

Diffusion in solids is a cooperative process. The migration of an impurity within a lattice formed by other atoms can introduce elastic stresses in the system, which increases the overall free energy of the system and reduces the further diffusion of the impurity. This effect should be quite small in the current systems because the lattice constants of Ni and 304 SS are pretty similar, where the constants of them are 0.350 and 0.352 nm, respectively. As a consequence, the influence of the aforementioned elastic stresses on the diffusion is neglected in the present study.

**Figure 3:**
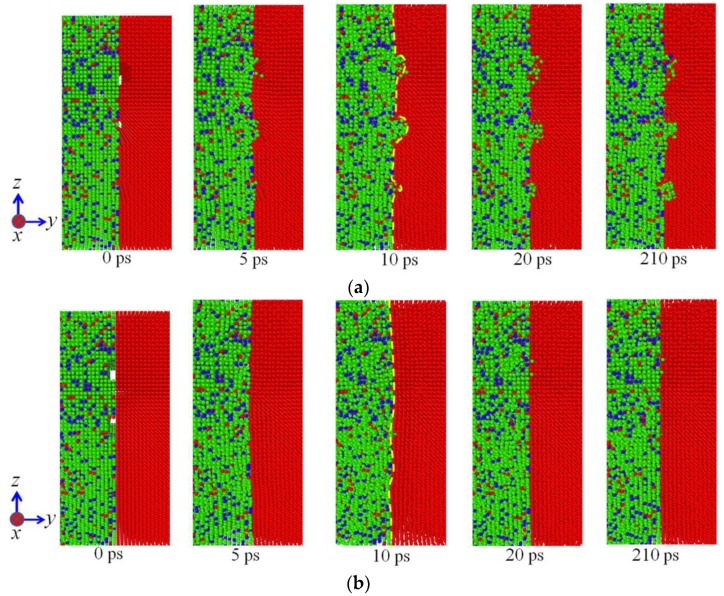
Evolution of diffusion configurations subjected to the vertical pressure of 50 MPa at 1200 K: (**a**) Model Ι; (**b**) Model ΙΙ.

Figure 3 illustrates the evolution of diffusion configurations subjected to the vertical pressure of 50 MPa at 1200 K, where only atoms near the interface are shown so as to present the diffusion behaviors clearly. It can be seen from Figure 3 that surface roughness has a significant influence on the diffusion behaviors of atoms during diffusion welding between two different materials. In the case of model Ι, the atoms in 304 SS gradually diffuse into the grooves in the pure Ni and finally the grooves are filled by these atoms. Meanwhile, the interface becomes uneven due to the diffusion. In the case of model ΙΙ, the grooves in 304 SS are mainly filled by the atoms in itself, which indicates that the atoms in pure Ni are difficult to diffuse into the 304 SS side during the diffusion welding. It can also be seen from Figure 3 that in the course of heating (prior to 10 ps), the interfaces of both the models are very uneven, as shown in the yellow dash lines of insets at 10 ps. This is due to the fact that the atoms near the grooves are mainly used to fill the grooves and atoms being far away from the grooves do not have enough time to diffuse to the regions lacking atoms. With the proceeding of temperature holding, the atoms being far away from the interface gradually diffuse toward the interface so as to maintain the balance between the two phases. According to the evolution of diffusion configurations of the two models, it can be concluded that the atoms in 304 SS are more active and they are more likely to diffuse during the diffusion welding between 304 SS and pure Ni. Consequently, in order to realize stronger connection between 304 SS and Ni, it is suggested that the rough surface should be set on the pure Ni rather than the 304 SS during the diffusion welding between them.

**Figure 4:**
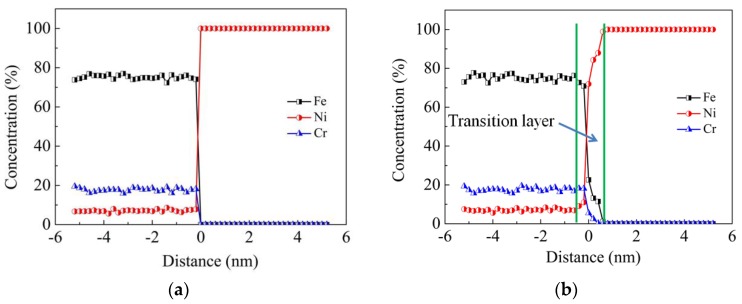

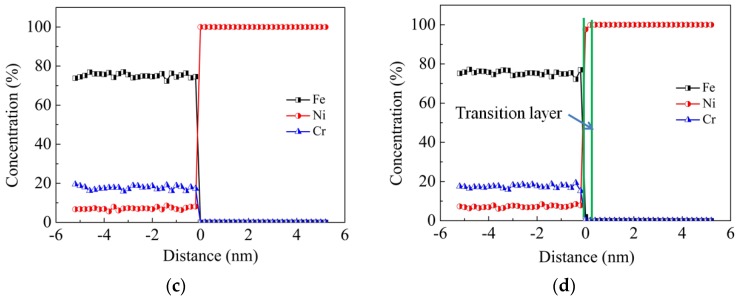
Concentration profiles of Fe, Ni and Cr elements along the vertical direction of interfaces at the temperature of 1200 K and the vertical pressure of 50 MPa: (**a**) Model Ι at 0 ps; (**b**) Model Ι at 210 ps; (**c**) Model ΙΙ at 0 ps; (**d**) Model ΙΙ at 210 ps.

Figure 4 illustrates the concentration profile of Fe, Ni and Cr elements along the vertical direction of interfaces at the temperature of 1200 K and the vertical pressure of 50 MPa. It can be found that in the transition layer, where concentrations vary considerably around the interface, the thickness of the Ni-rich phase is larger than that of the Fe-rich phase. This phenomenon indicates that more atoms of 304 SS diffuse into the Ni side and less Ni atoms diffuse into the 304 SS side. In other words, Fe and Cr atoms are more likely to diffuse. Comparing the concentration profiles of the two models, it can be seen that the transition layer of model ΙΙ is much thinner than that of model Ι. This is attributed to the fact that self-diffusion is dominant on the 304 SS side of model ΙΙ. The self-diffusion results from two conditions, where the first one is that the grooves on the 304 SS side provide spaces, and the second one is that Fe and Cr atoms diffuse more easily than Ni atoms, as has been demonstrated in Figure 3b. As a consequence, the roughness extent on the surfaces of different materials has a significant influence on the thickness of transition layer of diffusion welding.

**Figure 5:**
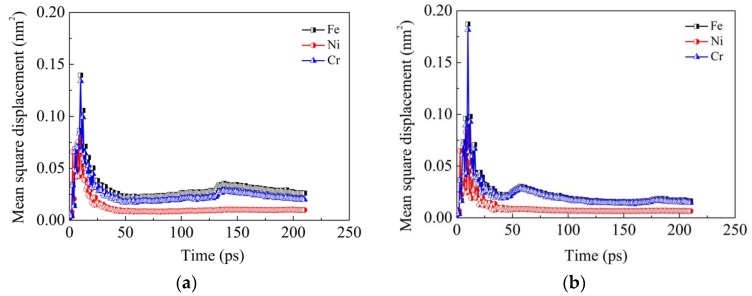
Mean square distance (MSD) results of Fe, Ni and Cr elements in the two models: (**a**) Model Ι; (**b**) Model ΙΙ.

In order to investigate the diffusion ability of each element during the current diffusion welding, MSD results of Fe, Ni and Cr elements in the two models are obtained, as shown in Figure 5. It can be seen that during the heating stage (prior to 10 ps), the MSD values of all the three elements increase sharply. However, during the holding stage (after 10 ps), these values drop down greatly at first and then vary very little during the remaining time. This phenomenon indicates that the heat is the driving force of atom diffusion and the diffusion is limited at a definite temperature. The decrease of diffusion ability at the later stage is due to the fact that the diffusion coefficient in the bulk material is less than the one on the surface. The initial fast diffusion mainly results from the surface atoms. When the surface atoms almost filled up the gap between the two sides and the grooves on the surface, they become one bulk material and the diffusion ability of the atoms in it is reduced. It can also be seen that additional second peaks exist in MSD curves of Fe and Cr elements in the two models. However, these second peaks occur at different times in the two models. These second peaks may be induced by the local creep deformation near the interface when the grooves are nearly filled up with the atoms near the grooves. At this moment, there exist vacancy defects in these regions due to the decrease of atom density. Under the combined action of external pressure and high temperature, creep deformation is easily induced in these regions. Meanwhile, the local creep deformation near the interface results in rigid movement of atoms far away from the interface. As a consequence, the MSD increases in a short time, which leads to the second peaks. The second peaks occur at different times in the two models due to the fact that the grooves in the model ΙΙ are filled up prior to those in the model Ι. The aforementioned results are consistent with Derby’s theoretical model [24,25], where power-law creep deformation occurs in the bridge of the interface after a period of surface diffusion. In addition, it can also be found from Figure 5 that the MSD values of Fe and Cr elements are both much larger than that of the Ni element, which further demonstrates the above conclusion that Fe and Cr elements diffuse more easily than the Ni element.

For the purpose of studying the influence of temperature and vertical pressure on the diffusion behaviors during diffusion welding between 304 SS and pure Ni, only model Ι was considered in the following sections.

**Figure 6:**
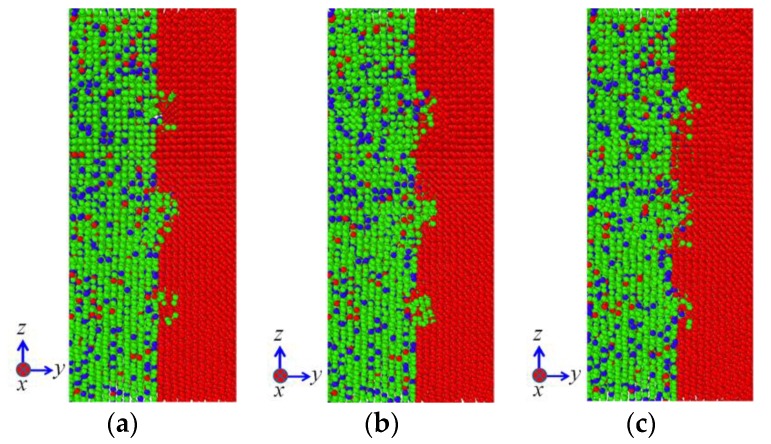
Influence of temperature on the diffusion configuration of model Ι subjected to the vertical pressure of 50 MPa: (**a**) 1000 K; (**b**) 1200 K; (**c**) 1400 K.

Figure 6 illustrates the influence of temperature on the diffusion configuration of model Ι subjected to the vertical pressure of 50 MPa. It can be noted that the temperature plays an important role in the interface diffusion of diffusion welding. With the increase of temperature, more and more atoms on the 304 SS side diffuse into the Ni matrix and the diffusion distances of Fe and Cr elements increase. In addition, a small amount of Ni atoms on the Ni side diffuse into 304 SS side as well, as shown in Figure 6c. It can be also seen from Figure 6c that the diffusion distances of Fe and Cr atoms are larger than those of Ni atoms. The aforementioned phenomena are attributed to the fact that the melting points of Fe and Cr (1811 and 1930 K, respectively) are both higher than that of Ni (1726 K), which indicates that both the bonds between Fe atoms and the ones between Cr atoms are stronger than those between Ni atoms. As the fraction of Fe and Cr elements is dominant in the 304 SS, the melting point of 304 SS is influenced little by the Ni element and hence it is higher than that of pure Ni. Consequently, the defects such as vacancies are induced more easily in the Ni matrix. Furthermore, the diffusion ability of Fe and Cr atoms is much higher than that of Ni atoms. In addition, grooves exist on the surface of Ni. As a result, the diffusion from 304 SS to pure Ni is dominant in the current investigation. It is well known that the diffusion coefficients for metallic self-diffusion or for diffusion between two dissimilar metals vary with temperature according to the Arrhenius type equation [26]

(4) $$D=D_{0}\exp\left(-\frac{Q}{RT}\right)\;$$

where *D*_0_ and *Q* are temperature independent constants, *R* the universal gas constant (8.314 J·mol^−1^·K^−1^), and *T* is the absolute temperature.

Based on Equation (4), it can be seen that the diffusion between 304 SS and pure Ni increases with increasing temperature. As a consequence, the results in Figure 6 follow the Arrhenius type equation and the 304 SS-Ni system present higher diffusion ability at elevated temperatures.

**Figure 7:**
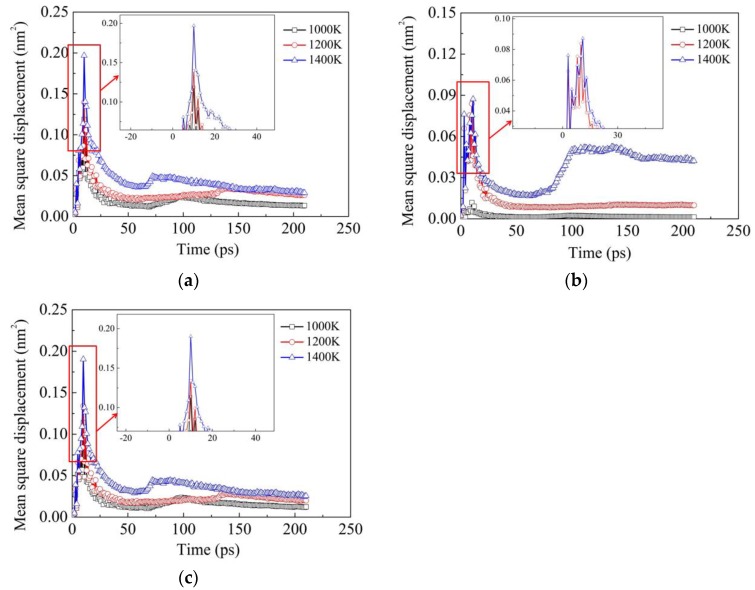
Influence of temperature on the MSD results of Fe, Ni and Cr elements in Model Ι subjected to the vertical pressure of 50 MPa: (**a**) Fe; (**b**) Ni; (**c**) Cr.

Figure 7 illustrates the influence of temperature on the MSD results of Fe, Ni and Cr elements in Model Ι subjected to the vertical pressure of 50 MPa. It can be seen that in general, the MSD values of all the three elements increase with increasing temperature, which demonstrates the above conclusion that the diffusion ability of two dissimilar metals increases with increasing temperature. Comparing the MSD values of Fe, Ni and Cr elements, it can be noted that the MSD values of Fe and Cr elements are much higher than those of the Ni element, which is also in accordance with the above interpretation that the diffusion distances of Ni atoms are smaller than those of Fe and Cr atoms.

**Figure 8:**
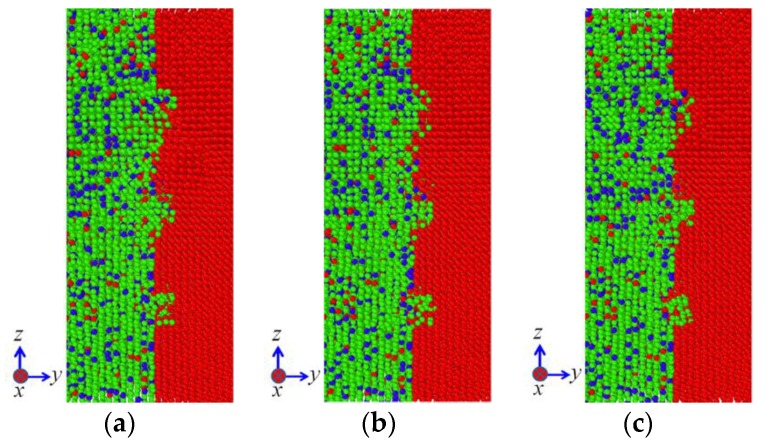
Influence of vertical pressure on the diffusion configuration of model Ι at 1200 K: (**a**) 0.5 MPa; (**b**) 5 MPa; (**c**) 50 MPa.

**Figure 9:**
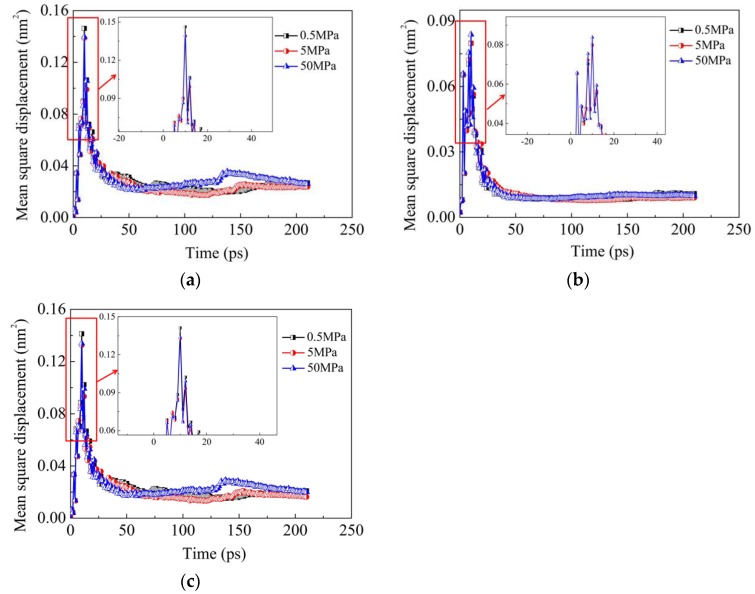
Influence of vertical pressure on MSD results of Fe, Ni and Cr elements in Model Ι at 1200 K: (**a**) Fe; (**b**) Ni; (**c**) Cr.

Figure 8 and Figure 9 illustrate the influence of vertical pressure on the diffusion configuration and the MSD results of Fe, Ni and Cr elements in model Ι at 1200 K, respectively. It can be seen from the two figures that the vertical pressure has a very weak influence on the diffusion behaviors of the interface. As shown in Figure 9, during a period of the diffusion welding, the MSD values of the three elements in the condition of 50 MPa are even lower than the ones in the conditions of lower vertical pressures, which indicate that the vertical pressure does not always have a positive influence on the diffusion acceleration of 304 SS-Ni system. Li et al. [27] found that the pressure can not only promote the void shrinkage in the interface region, but also facilitate the atomic diffusion across the bond line during their investigation on diffusion bonding of Ti-33Al-3V/TC17. However, in the present study, only the former phenomenon (groove shrinkage) is observed, while the latter case (atomic diffusion across the bond line) is not found. Indeed, the crucial role of the vertical pressure during diffusion welding lies in its ability of increasing the contact area via deforming the asperities or grooves at the interface, which provides more opportunities for diffusion between the two materials [23]. For a given contact area, the diffusion ability is mainly determined by the temperature and duration time. In addition, the applied pressure usually reduces the mobility of atoms within a lattice. Under the combination of positive and negative effects of the pressure, the vertical pressure has little influence on diffusion ability of the same model. The above conclusion can be demonstrated by Figure 9, where the values of the MSD curves under the three pressures are very close compared with those in Figure 7. As a consequence, it can be concluded that the pressure effect is smaller than temperature effect during diffusion welding between 304 SS and pure Ni.

## 4. Conclusions

(1) The roughness has a significant influence on the diffusion behaviors of atoms during diffusion welding between two different materials. During the diffusion welding between 304 SS and pure Ni, the atoms in 304 SS are more active and more likely to diffuse. As a consequence, in order to realize stronger connection between 304 SS and Ni, it is suggested that the rough surface should be set on the pure Ni rather than the 304 SS during the diffusion welding between them.

(2) Temperature plays an important role in the interface diffusion during diffusion welding between 304 SS and pure Ni. With the increase of temperature, the number of atoms diffusing to the opposite side increases and the diffusion distances increase as well. This is attributed to the fact that more defects such as vacancies are induced, which provides more opportunities for the atoms to diffuse into the opposite sides. As a consequence, the diffusion welding should be performed at an elevated temperature in the suitable temperature range.

(3) The influence of vertical pressure on the diffusion bonding between the two materials includes two aspects. One is to increase the contact area via deforming the asperities or grooves at the interface, which provides more opportunities for the diffusion between the two materials. The other is to reduce the mobility of atoms within a lattice. As a consequence, the pressure effect is smaller than the temperature effect during diffusion welding between 304 SS and pure Ni.

## References

[1] Kolmogorov V.L., Zalazinsky A.G. (1998). On metal joining and the prediction of the strength of solid-phase joints. J. Mater. Process. Technol..

[2] Miriyev A., Barlam D., Shneck R., Stern A., Frage N. (2014). Steel to titanium solid state joining displaying superior mechanical properties. J. Mater. Process. Technol..

[3] Rieth M. (2009). Diffusion weld study for Test Blanket Module fabrication. Fusion Eng. Des..

[4] Ustinov A.I., Falchenko I.V., Melnychenko T.V., Petrushynets L.V., Liapina K.V., Shishkin A.E. (2017). Diffusion welding through vacuum-deposited porous interlayers. J. Mater. Process. Technol..

[5] Jafarian M., Khodabandeh A., Manaf S. (2015). Evaluation of diffusion welding of 6061 aluminum and AZ31 magnesium alloys without using an interlayer. Mater. Des..

[6] Xua B., Wu X., Lei J., Cheng R., Ruan S., Wang Z. (2015). Laminated fabrication of 3D micro-electrode based on WEDM and thermal diffusion welding. J. Mater. Process. Technol..

[7] Aboudi D., Lebaili S., Taouinet M., Zollinger J. (2017). Microstructure evolution of diffusion welded 304L/Zircaloy_4_ with copper interlayer. Mater. Des..

[8] Azizi A., Alimardan H. (2016). Effect of welding temperature and duration on properties of 7075 Al to AZ31B Mg diffusion bonded joint. Trans. Nonferr. Met. Soc. China.

[9] Soltani S., Abdolrahim N., Sepehrband P. (2017). Molecular dynamics study of self-diffusion in the core of a screw dislocation in face centered cubic crystals. Scr. Mater..

[10] Terentyev D., Monnet G., Grigorev P. (2013). Transfer of molecular dynamics data to dislocation dynamics to assess dislocation–dislocation loop interaction in iron. Scr. Mater..

[11] Borodin E.N., Mayer A.E. (2015). Structural model of mechanical twinning and its application for modeling of the severe plastic deformation of copper rods in Taylor impact tests. Int. J. Plast..

[12] Wang B. (2013). Urbassek, H.M. Molecular dynamics study of the a–c phase transition in Fe induced by shear deformation. Acta Mater..

[13] Evteev A.V., Levchenko E.V., Belova I.V., Murch G.E. (2011). Molecular dynamics simulation of diffusion in a (110) B2-NiAl film. Intermetallics.

[14] Lu T., Niu G., Xu Y., Wang J., An Z., Liu H., Zhou H., Ding F., Luo G., Li X. (2016). Molecular dynamics study of the diffusion properties of H in Fe with point defects. Fusion Eng. Des..

[15] Bai D., Sun J., Chen W., Du D. (2016). Molecular dynamics simulation of the diffusion behaviour between Co and Ti and its effect on the wear of WC/Co tools when titanium alloy is machined. Ceram. Int..

[16] Song C., Lin T., He P., Jiao Z., Tao J., Ji Y. (2014). Molecular dynamics simulation of linear friction welding between dissimilar Ti-based alloys. Comput. Mater. Sci..

[17] Béjaud R., Durinck J., Brochard S. (2018). Twin-interface interactions in nanostructured Cu/Ag: Molecular dynamics study. Acta Mater..

[18] Plimpton S. (1995). Fast parallel algorithms for short-range molecular dynamics. J. Comput. Phys..

[19] Bonny G., Castin N., Terentyev D. (2013). Interatomic potential for studying ageing under irradiation in stainless steels: The FeNiCr model alloy. Model. Simul. Mater. Sci. Eng..

[20] Zhang M.X., Huang H., Spencer K., Shi Y.N. (2010). Nonomechanics of Mg–Al intermetallic compounds. Surf. Coat. Technol..

[21] Zuruzi A.S., Li H., Dong G. (1999). Effects of surface roughness on the diffusion bonding of Al alloy 6061 in air. Mater. Sci. Eng. A.

[22] Chu Y., Li J., Zhu L., Tang B., Kou H. (2017). Characterization of the interfacial-microstructure evolution and void shrinkage of Ti-22Al-25Nb orthorhombic alloy with different surface roughness during diffusion bonding. Intermetallics.

[23] Shao X., Guo X., Han Y., Lu W., Qin J., Zhang D. (2015). Characterization of the diffusion bonding behavior of pure Ti and Ni with different surface roughness during hot pressing. Mater. Des..

[24] Derby B., Wallach E.R. (1982). Theoretical model for diffusion bonding. Met. Sci..

[25] Derby B., Wallach E.R. (1984). Diffusion bonding: Development of theoretical model. Met. Sci..

[26] Claire D.L. (1953). The theory of D_0_ in the Arrhenius equation for self-diffusion in cubic metals. Acta Metall..

[27] Li H., Yang C., Sun L., Li M. (2017). Influence of pressure on interfacial microstructure evolution and atomic diffusion in the hot-press bonding of Ti-33Al-3V to TC17. J. Alloys Compd..

